# Phenotypic integration of brain size and head morphology in Lake Tanganyika Cichlids

**DOI:** 10.1186/1471-2148-14-39

**Published:** 2014-03-04

**Authors:** Masahito Tsuboi, Alejandro Gonzalez-Voyer, Niclas Kolm

**Affiliations:** 1 Department of Animal Ecology, Evolutionary Biology Centre, Uppsala University, Norbyvägen 18D, 75236 Uppsala, Sweden; 2 Department of Integrative Ecology, Estación Biológica de Doñana – Consejo Superior de Investigaciónes Científicas (EBD–CSIC), Av Américo Vespucio s/n, 41092 Sevilla, Spain; 3 Department of Zoology, Stockholm University, Svante Arrhenius väg 18B, 10691 Stockholm, Sweden

**Keywords:** Phenotypic integration, Geometric morphometrics, Phylogenetic comparative analysis, Lake Tanganyika cichlid, Brain evolution, Constraints

## Abstract

**Background:**

Phenotypic integration among different anatomical parts of the head is a common phenomenon across vertebrates. Interestingly, despite centuries of research into the factors that contribute to the existing variation in brain size among vertebrates, little is known about the role of phenotypic integration in brain size diversification. Here we used geometric morphometrics on the morphologically diverse Tanganyikan cichlids to investigate phenotypic integration across key morphological aspects of the head. Then, while taking the effect of shared ancestry into account, we tested if head shape was associated with brain size while controlling for the potentially confounding effect of feeding strategy.

**Results:**

The shapes of the anterior and posterior parts of the head were strongly correlated, indicating that the head represents an integrated morphological unit in Lake Tanganyika cichlids. After controlling for phylogenetic non-independence, we also found evolutionary associations between head shape, brain size and feeding ecology.

**Conclusions:**

Geometric morphometrics and phylogenetic comparative analyses revealed that the anterior and posterior parts of the head are integrated, and that head morphology is associated with brain size and feeding ecology in Tanganyikan cichlid fishes. In light of previous results on mammals, our results suggest that the influence of phenotypic integration on brain diversification is a general process.

## Background

Brain size is highly variable among vertebrates [1,2]. This variation is often considered to be affected by adaptations to the physical and social environment through natural selection operating on brain size [3-6]. However, adaptation to a specific cognitive environment is not the only source of variation and various other factors could also play an important role in shaping contemporary diversity in brain size [2]. Organisms need their parts to be integrated in order to function as a coherent whole (i.e. phenotypic integration [7,8]). Brain morphology (i.e. size and shape) is developmentally and genetically integrated with skull morphology [9-12]. In addition, the ventral part of the skull is integrated with jaw morphology, which in turn is strongly affected by feeding ecology [13,14]. As a consequence of skull-brain and skull-jaw integrations, feeding adaptations may conflict with brain size evolution [12,15-18]. At the same time, the degree of integration is not necessarily uniform throughout the entire organism. Certain groups of traits often form subunits, or modules, that are relatively independent of each other [19]. Whether the integration of different parts of the head influences brain size depends on the interaction between integration and modularity, because an increased level of integration constrains evolutionary changes while an increased level of modularity allows traits to evolve independently [20-23]. Hence, investigation of the degree of integration and modularity is important to understand the intrinsic morphological adaptations and constraints that affect vertebrate brain evolution. In particular, such analyses should target organisms with high levels of variation in both head and brain morphology.

The cichlids of Lake Tanganyika are an interesting model group to investigate the integration between skull and brain because of their remarkable diversity in both brain size and head morphology. The relative brain size (i.e. brain size controlling for body size allometry) of Tanganyikan cichlids is correlated with several ecological and social factors such as diet [6,24], habitat complexity [24,25], and parental care patterns [6]. Also the sizes of separate brain regions covary with ecological variation. For instance, the relative size of the telencephalon and cerebellum have been shown to increase as environmental complexity increases while the size of the olfactory bulb and the dorsal medulla decrease [25,26], and the optic tectum is negatively correlated with the depth at which the species live [24,26]. Overall, complex intra-and interspecific interactions of the cichlid community in the shallow rocky habitat of the lake [27-29] may have selected for large brains in algae-eating littoral species [6]. Tanganyikan cichlids are also known as one of the few families among vertebrates with extreme variation in body shape [30]. The largest component of overall shape variation is concentrated in the facial region of the head [31], which is strongly influenced by the supraoccipital crest morphology of the skull [32]. Moreover, the head morphology of Tanganyikan cichlids shows a tight correspondence with diet [32-37]. The cichlid fishes from Lake Tanganyika thus offer an interesting opportunity to test the hypothesis that phenotypic integration may link eco-morphological adaptation and brain size evolution.

The high degree of integration between the different parts of the head is widely reported across vertebrates (laboratory mice [38-40], domestic dogs [41], carnivores and marsupials [42], birds [43], *Anolis* lizards [44]). Interestingly, previous studies of the integration and modularity of the different parts of the head in East African cichlids have yielded somewhat conflicting results. Two studies have found that jaw and skull morphology are genetically correlated in support of phenotypic integration (28,29). However, a recent comparative study proposed two independent modules in the head of cichlids [45]. These are the pre-orbital module that encompasses the upper and lower jaws, and the post-orbital module that encompasses the posterior parts of the skull and the operculum [45]. Hence, whether the head shape of cichlid represents a relatively integrated unit, or consists of several independent modules remains an open question. At the same time it is critical to address this issue when investigating the link between head and brain morphology. Integration of the entire head would result in natural selection acting on the feeding apparatus of cichlids [37] which would influence the rest of the head as well. If head morphology and brain size are also integrated, eco-morphological adaptations might indirectly affect brain size evolution [12,15]. Alternatively, if the different parts of the head represent independent modules, eco-morphological adaptation could occur without influencing other morphological aspects within the head. Under such modularity of the different parts of the head, we would not predict a strong association between eco-morphological adaptation and brain size evolution.

In this study, we use landmark-based geometric morphometric phylogenetic analyses to test for the existence of phenotypic integration between various aspects of head morphology and brain size in Lake Tanganyika cichlids. According to our hypothesis, we test two aspects of integration. First, we test if the head is composed of morphologically independent modules or if it represents an integrated morphological unit. We then investigate whether head morphology is integrated with brain size or brain region volumes while considering the potentially confounding effect of prey utilization patterns that may affect head morphology.

## Methods

### Data

We chose Lake Tanganyika cichlids as our study group because they present the greatest shape variation among the cichlids of the African Great Lakes [30]. Our sample included 166 individuals across 35 species, representing 9 of the 12 tribes to which the Lake Tanganyika cichlids have been assigned [46]. Samples were all wild caught sexually mature individuals. Intraspecific sample sizes ranged between 3–7 individuals, except for one species (*Benthochromis tricoti*) for which we had two specimens (data is available as online Additional file 1: S1). To test for the potentially confounding effect of large within-species variation, particularly in light of sexual dimorphism in brain size, we performed an ANOVA with species and sex as factors on a data-set with matching numbers of male and female samples in each species. The analysis showed that interspecific variation in brain weight (Ssq = 5.45, *F*_29_ = 28.97, *p* < 0.001) was much higher than within-species between-sex variation (Ssq = 0.001, *F*_1_ = 0.95, *p* = 0.76) and the interaction between species and sex (Ssq = 0.18, *F*_29_ = 0.95, *p* = 0.54). Our data should thus be robust against within-species sex differences. Specimens were sacrificed using an overdose of benzocaine. After measuring standard length and head width (the distance between right and left of the dorsal end of operculum), the head was severed and preserved in 4% paraformaldehyde in a phosphate buffer for tissue fixation and preservation. Whole brains were obtained from dissected heads following fixation and weighed using a Precisa 125A electronic scale (precision = 0.01 mg; Precisa Instruments AG, Switzerland). All cranial nerves, optic nerves and meningeal membranes were removed and the brain was severed from the spinal cord just posterior of the dorsal medulla. Since brain volume and brain weight are highly correlated in our dataset (*r* = 0.96, [26]), we used brain weight as a proxy of brain size. We also collected information on feeding mode because this is strongly associated with head morphology in cichlid fishes [32,36]. Feeding mode in cichlid fishes can roughly be divided into biting and suction [32,47]. To assign each species to either of these two categories, we first searched for a direct description of feeding behavior and assigned the feeding mode accordingly. Second, for those species for which we failed to assign the category by direct descriptions, we assessed the most likely feeding mode based on diet. Among the major food items consumed by Lake Tanganyika cichlids, gastropods and attached algae require manipulation by the jaw whereas zooplankton, phytoplankton, shrimp and fish are taken through suction [32]. Therefore, we categorized a species as a suction-feeder if it was reported to feed on zooplankton, phytoplankton, shrimp and/or fish and as a bite-feeder if it was reported to feed on gastropods and/or attached algae. We used the following sources to obtain data for feeding behavior and food items: Liem [47], Yuma *et al.*[48], Konings [49,50], Yamaoka [51-53], Yuma and Kondo [54], Takamura [55], Takeuchi *et al.*[56], Ochi [57].

### Geometric morphometrics

**Figure 1:**
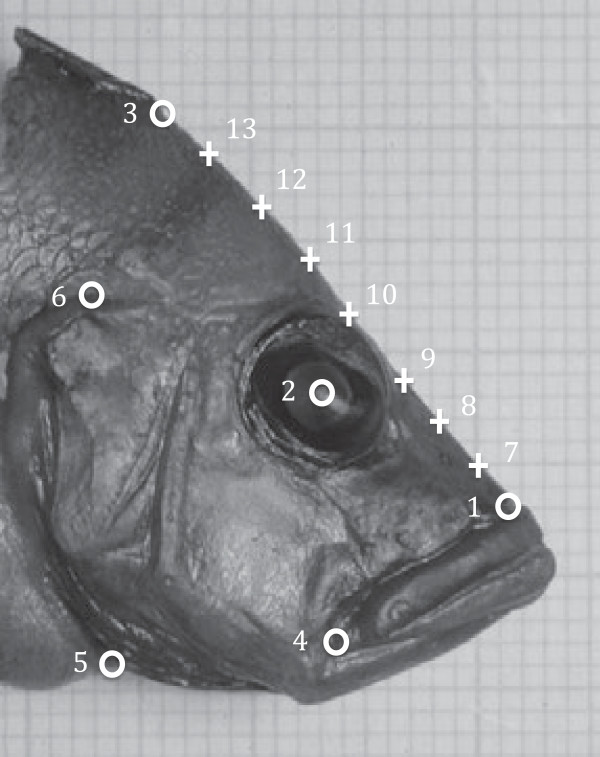
**Positions of the landmarks in our study:** Landmarks that are shown with a  symbol include: (1) anterior tip of the snout, (2) center of the eye, (3) anterior tip of the dorsal fin, (4) posterior border of upper lip, (5) posterior border of the branchiostegal membrane on the ventral midline, (6) anteriodorsal end of the gill cover. Seven internal semi-landmarks (7 to 13) between landmark one and three are also represented with a  symbol.

Images of the lateral sides of 156 individuals were taken with a reflex digital camera (Nikon D 70 with an AF Micro Nikkor 60 mm 1:2.8 D macro lens). Assuming symmetry, we used the best quality images either from the right or left side. A permutation test using Procrustes distance confirmed that the shape was not different between the photos of the separate sides (*p* > 0.62 in all cases). We measured the head length as the distance between the foremost point of the snout and the rearmost point along the operculum using a scale photographed in the background of the images. Using TpsDig version 2.16, we digitized six homologous landmarks and seven semi-landmarks along the edge of the forehead to capture the variation in forehead shape where the largest proportion of variation is concentrated [31] (Figure 1). We again tested the potential influence of sexual shape dimorphism in our analysis using MANOVA with the shape of each specimen as the response variable and species and sex as explanatory factors, and confirmed that interspecific difference in shape was much higher (Pillai’s Trace = 10.61, *F*_550, 2112_ = 3.58, *p* < 0.001) than intraspecific between-sex differences (Pillai’s Trace = 0.29, *F*_44, 152_ = 0.60, *p* = 0.97). Generalized Procrustes Analysis (GPA) was performed to mathematically remove the variation of position, size, and rotation of the landmark configurations [58]. Semi-landmark sliding was computed by minimizing summed procrustes distances between samples and the average shape. Because we were interested in relatively large-scale interspecific morphological variation, the choice of semi-landmark sliding methods should introduce only minor variation in our analyses [59]. We first performed GPA and semi-landmark sliding for individuals of each species and retained the species average shape. Subsequently, another GPA and semi-landmark sliding was performed with the average shape of 35 species, and the aligned species shape in a common shape space was retained for further investigations.

### Testing for integration and modularity of separate regions in the head

Using the previously proposed modules of the cichlid skull [45], we hypothesized that the pre-orbital and post-orbital parts of the head form two separate modules. Hence, the pre-orbital module included landmarks 1, 4, 7, 8, and 9 and the post-orbital module included landmarks 2, 3, 5, 6, 10, 11, 12, and 13 (Figure 1). The hypothesis of modularity predicts that the covariation between the landmarks in the pre-orbital part of the head and those in the post-orbital part of the head should not be higher than the covariation between randomly generated partitions of landmarks [19,60,61]. We quantified the strength of covariation between subsets of landmarks using the RV coefficient, which can be interpreted as a multivariate generalization of the bivariate *R*^*2*^ value [61]. To assess our hypothesis of modularity, we compared the RV coefficients for the partition of landmarks into pre-and post-orbital compartments with the distribution of the RV coefficients for randomly generated partitions. Randomized partitions were created by randomly selecting the same number of landmarks as those in the pre-and post-orbital modules (i.e. five and eight landmarks). We allowed randomized partitions to include only spatially contiguous landmarks [61]. We used MorphoJ ver. 1.06 [62] to test for modularity.

Prior to running the analyses, we formally assessed if the shape variables presented phylogenetic signal using a multivariate estimate of the *λ* parameter in the package phytools [63] in R version 2.13.2 [64]. *λ* = 0 denotes that traits evolve independent of the phylogeny while *λ* = 1 denotes that traits of any pair of species covary in direct proportion to the distance along the phylogeny to their nearest common ancestor [65]. A transformation of the phylogenetic tree based on the multivariate *λ* estimate would then allow us to use independent contrasts and obtain the same result as under a phylogenetic generalized least squares method [66,67]. Using a molecular phylogeny reconstructed with mitochondrial sequences downloaded from Genbank (for details on the phylogeny reconstruction, see [6]), the maximum likelihood estimate of the multivariate *λ* < 0.001, which indicates that shape is highly labile and presents very little phylogenetic signal. Under such circumstances, it is justified to test our modularity hypothesis without phylogenetic correction, as the results of phylogenetic and non-phylogenetically controlled analyses will converge.

### Size correction

In order to control for the allometric effect of body size on brain weight, we used phylogenetic principal component analyses (PPCA) [68]. Prior to size correction, brain weight, standard length, head length, and head width were log-transformed. Considering the large variation in the relative size of different parts of the body among cichlid species [32], we performed PPCA on three variables normally used to describe body size in fishes: standard length, head width, and head length, and retained the first principal component (loading; standard length 0.86, head width 0.97, head length 0.80) as a generic size measure (referred to as body size hereafter). Subsequently, we obtained body size corrected brain weight by using a phylogenetic size correction (PSC) that computes the residual, i.e. the deviation between the estimated value from a least squares regression and the data while controlling for phylogenetic non-independence [68]. Bivariate correlations revealed that head shape (i.e. the first four principal components for overall shape) and body size were not correlated (*R*^*2*^; 0.05 (PC1), 0.08 (PC2), 0.005 (PC3), 0.003 (PC4)), suggesting the use of residuals would not introduce bias in the analysis [69]. We performed PSC on brain weight against body size, and retained phylogeny-corrected residuals as a proxy of relative brain size (referred to as brain size hereafter). The phylogenetic signal was high in both PPCA (*λ* = 0.95) and PSC (*λ* = 1), indicating the necessity for phylogenetic correction. PPCA and PSC were performed using the phytools package [63] in R version 2.13.2 [64].

### Phylogenetic comparative analyses of phenotypic integration between head morphology and brain size under the influence of feeding ecology

Our assessment of covariation between head morphology and brain size, feeding mode, and body size involved testing for correlated evolution between two sets of several variables. In order to incorporate such a multivariate model while taking the phylogenetic non-independence into account, we performed Phylogenetic Generalized Least-Squares [70-72] in a multivariate framework (mPGLS) [73]. Prior to mPGLS, we reduced the number of variables, to obtain a robust result given the sample size, using principal component analysis (PCA) on the overall shape variables. Using MorphoJ ver. 1.06a [62], we performed a non-phylogenetic PCA because our shape data did not show phylogenetic signal (*λ* < 0.001). Following Horn’s parallel analysis [74,75] as recommended by Monteiro [76], we retained the first four principal components that cumulatively explained 84.56% of the total shape variance (PC1: 35.75%, PC2: 31.32%, PC3: 10.65%, PC4: 6.85%). Subsequently, we assessed the phylogenetic signal of our multivariate model with the first four principal components as the response matrix and body size, brain size, and feeding mode as predictor variables using the phytools package [63], and found *λ* = 1 for the residuals of the model. Then, we performed mPGLS with *λ* = 1, i.e. a Brownian motion model, to test the integration between head shape and brain size. We also tested the robustness of our test using overall brain size against structural heterogeneity of the brain by performing mPGLS on size of the six major brain regions (i.e. olfactory bulb, telencephalon, optic tectum, cerebellum, hypothalamus, and dorsal medulla) as a predictor variable and head shape (PC1-4) as a response matrix, using overall brain weight to control for the effect of size (see Additional file 1: S1 for details of the analysis). R version 2.13.2 [64] was used to perform mPGLS and Horn’s parallel analysis.

## Results

**Figure 2:**
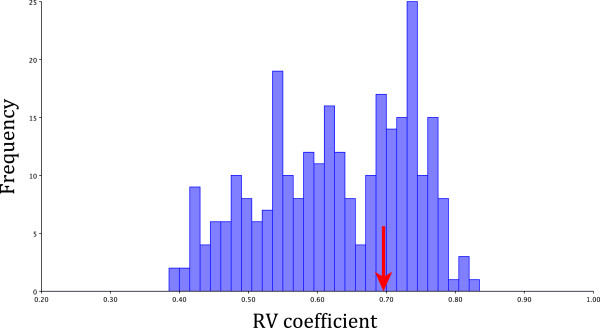
**Analysis of modularity: graph shows a histogram of the RV coefficients between 279 random partitions of the head of Lake Tanganyika cichlids.** The value of the RV coefficient between our hypothetical modules (pre and post-orbital modules, RV coefficient = 0.70) is indicated with an arrow.

In our sample of Lake Tanganyika cichlids, the RV coefficient between pre-orbital and post-orbital landmarks was 0.70 (Figure 2). This value suggests relatively high integration of overall head shape in Tanganyikan cichlids. 181 RV coefficients out of 279 randomly generated partitions were lower than the RV coefficient from our *a priori* hypothesis (Figure 2), indicating that the covariation between the pre-orbital and post-orbital parts of the head was higher than expected by chance. Overall, our results are not in line with those of a previous comparative study on cichlid fishes which suggested that the anterior and posterior parts of the head represent separate modules [45].

**Figure 3:**
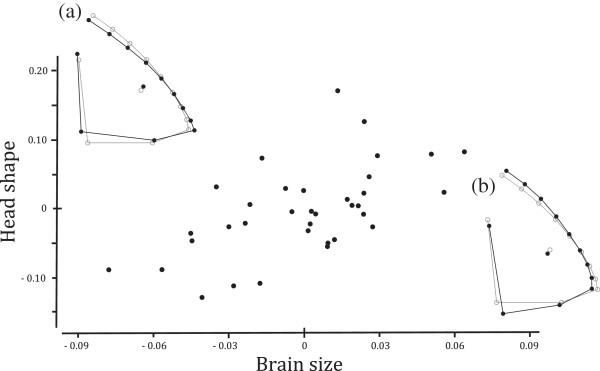
**Relationship between brain size and head shape: the horizontal axis represents the brain size and the vertical axis represents the regression scores of head shape.** To visualize the result, **(a)** head shape corresponds to small brain size (−0.8) and **(b)** head shape corresponds to large brain size (0.8) are presented by black lines with closed circles. Consensus configuration is also presented with grey lines with open circles. The graph was produced using MorphoJ ver. 1.06a [62] with procrustes coordinate as a response matrix and brain size, body size as covariates and grouped by feeding mode. Please note that this figure was made without phylogenetic corrections, for the purpose of visualization only.

**Figure 4:**
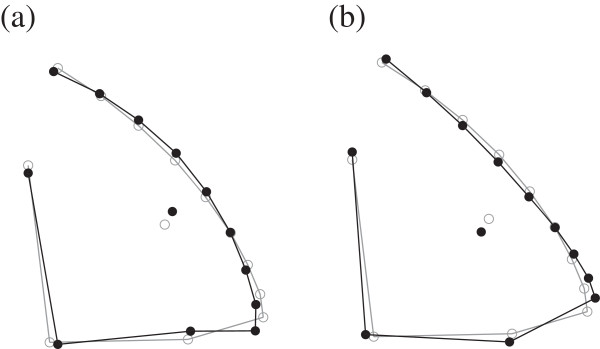
**The consensus configuration of the two groups of different feeding modes: the black lines with closed circles to the left represent the average shape of bite feeder (a), and suction feeder (b) to the right. **The grey lines with open circles represents the consensus configuration of all 35 species in our study.

The associations between head shape (the first four principal components of shape variables) and the three independent variables (body size, brain size, feeding mode) are summarized in Table 1. We found that both feeding mode (Pillai’s trace = 0.35, Approx. *F*_*4,28*_ = 3.70, *P* = 0.02) and brain size (Pillai’s trace = 0.43, Approx. *F*_*4,28*_ = 5.26, *P* = 0.002) were correlated with head shape. The direction of the shape change associated with brain size is presented in Figure 3. A dorso-ventrally higher head shape (Figure 3b) was associated with a larger brain, while a more snout-elongated head shape (Figure 3a) was associated with a smaller brain. The average head shapes for bite feeders and suction feeders are presented in Figure 4. Bite feeders had a more downward-pointing mouth and a curved forehead (Figure 4a), while suction feeders had a more upward-pointing mouth and a relatively straight forehead (Figure 4b). Finally, we found that none of the volumes of the six major brain regions were significantly associated with head shape (online Additional file 1: S1 and Table S1).

**Table 1:** Phylogenetic generalized least square multivariate regression models (mPGLS)

	**Multivariate PGLS**				
**Predictor**	**Pillai’s trace**	**Approx. *F***	**d.f. num.**	**d.f. den.**	***P***
Body size	0.21	1.91	4	28	0.14
Brain size	0.43	5.26	4	28	**0.002**
Feeding mode	0.35	3.70	4	28	**0.02**

Response variables are principal component 1, 2, 3, and 4 after principal component analysis on all shape variables (see text for details). Predictor variables are body size, brain size, and feeding mode. Significant relationships are presented in bold font.

## Discussion

Our study represents one of the first macro-evolutionary studies of phenotypic integration between brain size and head morphology in non-mammalian vertebrates and several insights can be gained from our results. First, we found support for phenotypic integration rather than modularity between the pre-orbital and post-orbital parts of the head. Second, head morphology was linked to prey utilization. Third, when controlling for the association between food utilization and head morphology, we found that brain size in Lake Tanganyika cichlids was closely associated with variation in head morphology. Together, these findings indicate that brain evolution and trophic adaptations may interact through phenotypic integration of brain size and head shape.

### Phenotypic integration and modularity of separate regions in the head

Our test for modularity did not support the existence of independent modules within the head. Our result therefore disagrees with a previous comparative analysis which suggested the existence of independent modules within the head of East African cichlids [45]. We propose four possible reasons for the disagreement between our results and those of Parsons *et al.*[45]. First, we had a quite different selection of species in our dataset. For instance, Parsons *et al.*[45] included scale-eating species (*Perissodini*) in their dataset while we did not. Second, our choice of landmarks and semi-landmarks are slightly different from Parsons *et al.*[45]. However, given the remarkable diversity in head shape of Tanganyikan cichlids [30,31], differences in the choice of landmarks should have only minor effects on the results. Third, our test of modularity is based on RV coefficients between blocks of landmarks [61], while Parsons *et al.*[45] investigated modularity using an approach based on a goodness of fit statistic, γ [77]. It is therefore possible that our analyses differ in their abilities to unveil patterns of integration and modularity. However, it is also important to note that integration and modularity refer to relative measures between two extremes of a continuum, total integration and total modularity, rather than refer to two discrete states [19,23,60]. Hence, it might be possible that the pre- and post-orbital parts of the head represent some degree of simultaneous integration and modularity. Fourth, we assessed the strength of phylogenetic signal in our data prior to our analysis while Parsons *et al.*[45] did not consider phylogenetic non-independence. Our data showed no phylogenetic signal. However, since the species selection in our dataset was quite different from that of Parsons *et al.*[45], it is possible that the lack of phylogenetic correction could have affected their results. Isolated or in concert, these factors may have affected the difference in the results between our analysis and the previous analysis.

Instead, our results indicated phenotypic integration between the pre-orbital parts (which included the skull) and the post-orbital parts (which included the upper and lower jaws) of the head. Given the close association between forehead shape and skull shape [32] this integration could be interpreted as a manifestation of the close functional and genetic integration between the skull and the jaws [78,79]. The neurocranium (i.e. skull) and the maxillae are connected by articulations through which biting force is transmitted during mouth opening and closing [32,79], and the angle of the anterior portion of the neurocranium (i.e. the vomer) is related to the capability of the neurocranium to resist biting force [80]. A steep vomerine angle therefore results in less force being concentrated at any specific point of the vomer, enabling the feeding apparatus to exert a strong biting force [79]. Consistent with these observations, a curved forehead with an obtuse snout angle is nearly ubiquitous among bite feeding cichlid species [32]. Moreover, the functional integration of the neurocranium and lower jaw has been demonstrated to coincide with a shared genetic basis, due to either genetic linkage between two functional loci or pleiotropy [78,79]. Our results, which support these previous micro-evolutionary patterns, suggest that the integration between the skull and the jaws has played an important role in forming the craniofacial diversity of Lake Tanganyika cichlids also at the macro-evolutionary level.

### Eco-morphological adaptation in Tanganyikan cichlids

Our results confirmed the widely reported evolutionary link between head morphology and feeding ecology in Lake Tanganyika cichlids [32-37]. The association we found here is in accord with the functional morphology of teleost fishes, where bite feeders require high and curved head profiles to produce strong biting force while suction feeders require a snout-elongated morphology that increases the velocity of the oral jaw and/or themselves towards the prey during the strike [32,37,81-84]. In addition to the functional morphology *per se*, a round forehead with a downward-pointing mouth are thought to be adaptations for grazing algae from the substrate [32]. However, the head profile must be designed so that it can accommodate and protect the brain, and the brain itself is subject to selection to fulfill various social and environmental cognitive demands [6,24-26].

### Phenotypic integration of brain size and head morphology

After controlling for the interaction between feeding mode and head morphology, our results confirmed a strong association between head morphology and brain size. We showed that species with a high head profile have larger brains while species with a more elongated head profile have smaller brains. Given that an elongated body shape is associated with a smaller head volume in cichlid fishes [85], our result could be interpreted as a consequence of spatial constraints acting on brain size. Alternatively, our results may indicate that selection to enlarge the brain requires coevolution of head shape to accommodate the necessary space in the brain cavity. The rocky littoral areas of Lake Tanganyika harbor a diverse species assemblage where complex species interactions are frequent [27]. Increased cognitive demands associated with interspecific territory defense [55] or group foraging [28] may have selected algae-eating littoral species to have relatively large brains [6,24-26]. Our result may therefore be a consequence of positive selection acting on increased brain size. However, cognitive adaptation and trophic adaptation are not necessarily mutually exclusive. Given the high energetic costs of developing and maintaining neural tissue [86-88], it is unlikely that large brains evolve simply because they have enough physical space [89]. On the other hand, considering the significance of outer morphology for various aspects of ecological performance [82,90], head morphology is also less likely to evolve merely because space for the brain is necessary. Therefore, we propose that multiple facets of phenotypic integration of brain size and head morphology operate simultaneously in forming the diversity of brain size in Lake Tanganyika cichlids. For instance, trophic adaptation may influence brain size evolution by biasing the evolutionary rate [91-93] while adaptations for better cognitive ability may simultaneously increase brain size [88]. Morphological integration may also arise from developmental associations of skull tissue and brain tissue that are generated from the same cell lineage [94,95]. Clearly, understanding the exact processes through which the phenotypic integration of brain size and body morphology may work at the macro-evolutionary level will require further work.

## Conclusions

Our multivariate phylogenetic comparative study found support for integration of the head morphology of Lake Tanganyika cichlids, and that evolutionary associations exist among head morphology, feeding mode, and brain size. Our study therefore indicates that head-brain phenotypic integration might have played an important role in forming the macro-evolutionary variation of brain size in cichlid fishes, a group which has been an important model system for the study of phenotypic diversification. Given the strong and general association of ecology and head shape in teleost fishes [32-36], our results indicate that eco-morphological adaptation and cognitive adaptation can interact through phenotypic integration of head morphology and brain size. Specifically, the head morphology associated with suction feeding corresponds to smaller brains, suggesting that ecological adaptation to a piscivorous life style might have sacrificed encephalization. Identifying the factors that promote and constrain brain evolution is fundamental to understanding the processes behind vertebrate brain diversification. Eco-morphological adaptation could be a critical source of brain diversification also in many other groups of vertebrates, such as carnivorous mammals [12,16], and hominids [15]. Given the fact that head morphology is also a key ecological adaptation in birds [96-98] and lizards [99-101], investigations in these groups would be interesting to further test the general influence of eco-morphological adaptation as a factor affecting vertebrate brain diversification.

## Competing interests

We declare that we have no competing interests.

## Authors’ contributions

All authors have read and approved the final manuscript. MT participated in the design of the study, carried out the analyses, and drafted the manuscript. AG-V conducted brain dissections and data collection, assisted in the analyses, and critically revised the manuscript. NK conceived the study, obtained funds, participated in the design of the study and helped to draft the manuscript.

## Supplementary Material

Additional file 1 Heterogeneity of the brain. Click here for file
