# Supplementary material for: Phenotypic integration of brain size and head morphology in Lake Tanganyika Cichlids
Source: BMC Evol Biol. 2014 Mar 4;14:39. doi: 10.1186/1471-2148-14-39 (PMC4015177; doi:10.1186/1471-2148-14-39)
Supplement: Additional file 1 — Heterogeneity of the brain. [file 1471-2148-14-39-S1.pdf]

Online supplementary Material S1

Heterogeneity of the brain

The vertebrate brain is composed of functionally distinctive structures [1]. To address if our found evolutionary association between brain size and head shape is influenced by structural heterogeneity of the brain, we ran multivariate phylogenetic generalized least square (mPGLS) using six major brain regions, olfactory bulb, telencephalon, optic tectum, medulla, cerebellum, and hypothalamus instead of overall brain size. We used volumetric data on these structures of our specimen measured by Gonzalez-Voyer and Kolm [2]. Following the statistical protocol of mPGLS described in the main text, we used first four principal components from the principal component analysis on head shape. Prior to mPGLS, we performed phylogenetic size correction (PSC) between one of six structures and the whole brain weight to control the effect of size. All variables are log transformed before PSC. Subsequently, we performed mPGLS on four principal components of head shape as a response matrix and one of six size-corrected brain regions as an explanatory variable. Table S1 summarizes the result of mPGLS. We found that none of six brain regional size was significantly correlated with head shape. These results suggest that the found association between brain size and head shape in our study is mainly driven by a component of brain regional size that evolves in concert with each other. Therefore, the usage of overall brain size in our study should not be biased by the structural heterogeneity of brain.

Table S1: Results of multivariate PGLS of six major brain regions as a predictor and principal component 1, 2, 3, and 4 after the principal component analysis on all shape variables as a response matrix.

	Multivariate PGLS				
Predictor	Pillai's trace	Approx. *F*	d.f. num.	d.f. den.	*P*
(a) Olfactory bulb	0.17	0.71	8	62	0.68
(b) Telencephalon	0.38	1.80	8	62	0.09
(c) Optic tectum	0.33	1.50	8	62	0.18
(d) Medulla	0.17	0.73	8	62	0.67
(e) Cerebellum	0.29	1.33	8	62	0.24
(f) Hypothalamus	0.39	1.92	8	62	0.07

References

1. Nieuwenhuyis R, ten Donkelaar HJ, Nicholson C: **The Central Nervous System of Vertebrates**: Springer; 1998.
2. Gonzalez-Voyer A, Kolm N: **Sex, Ecology and the Brain: Evolutionary Correlates of Brain Structure Volumes in Tanganyikan Cichlids**. *PLoS One* 2010, **5**(12):e14355.

Table S2. Information for 35 species used in our study. Sample size of each sex, average measures of standard length, brain weight, head length, and head width, feeding mode, diet, and references for diet and feeding mode are presented.

Species	Sample Size		Standard Length	Brain Weight	Head Width	Head Length	Feeding Mode	Diet	References
	Male	Female							
Altolamprologus compressiceps	1	5	84.50	69.67	9.09	25.83	suction	shrimp	Yuma and Kondo 1997
Aulonocranus dewindti	4	2	89.50	79.83	12.92	27.00	suction	invertebrates, crustaceans	Koning 2005
Benthochromis tricoti	0	2	146.00	116.50	14.64	39.00	suction	zooplankton	Koning 2005
Callochromis melanostigma	2	1	61.33	42.33	8.48	28.00	suction	benthic prey	Koning 2005
Callochromis pleurospilus	4	1	80.20	62.75	10.54	27.67	suction	benthic prey, invertebrate	Koning 2005
Ctenochromis horei	2	1	119.50	162.00	14.56	28.00	suction	benthic prey, fish, invertebrate	Koning 2005, Ochi 1993
Cyathopharynx furcifer	2	2	130.50	139.00	18.37	28.50	suction	aufwuchs	Yamaoka 1991
Cyphotilapia frontosa	2	3	160.80	205.20	23.08	23.67	suction	fish, invertebrate	Koning 2005
Cyprichromis leptosoma	4	2	90.50	77.17	10.39	29.40	suction	zooplankton, copepod	Koning 2005
Cyprichromis microlepidotus	5	0	117.80	117.40	13.70	36.00	suction	zooplankton	Koning 2005
Enantiopus melanogenys	3	1	147.50	107.50	15.91	20.50	suction	benthic prey	Koning 1998
Eretmodus cyanostictus	3	1	60.00	38.25	7.26	29.75	bite	algae	Yamaoka et al 1986, Yamaoka 1987
Gnathochromis permaxillaris	2	2	115.25	85.25	13.83	36.67	suction	benthic prey	Koning 1998
Greenwoodochromis christyi	1	2	115.00	98.67	14.33	33.67	suction	fish, invertebrate	Koning 2005
Haplotaxodon microlepis	4	2	99.67	92.33	12.04	25.33	suction	fish	Koning 2005
Julidochromis marlieri	1	5	81.50	47.00	9.65	21.00	bite	sponges, invertebrates	Koning 1998
Julidochromis regani	2	4	93.83	52.83	11.06	23.33	bite	sponges, invertebrates	Koning 1998
Lamprologus callipterus	4	0	85.25	49.25	10.15	21.67	suction	shrimp	Yuma and Kondo 1997
Lepidolamprologus nkambae	5	1	118.67	85.67	12.32	19.33	suction	small fish, invertebrate	Koning 2005
Limnotilapia dardennii	4	2	63.33	57.50	8.15	30.67	bite	algae	Yamaoka 1983
Neolamprologus brevis	2	2	44.25	24.00	6.21	10.00	suction	zooplankton	Koning 2005
Neolamprologus brichardi	1	6	71.86	36.00	8.72	18.00	suction	zooplankton	Koning 2005
Ophthalmotilapia boops	1	2	89.33	83.00	12.47	23.00	suction	aufwuchs	Yamaoka 1991
Ophthalmotilapia nasuta	2	2	92.25	91.00	13.03	26.25	suction	aufwuchs	Yamaoka 1991
Ophthalmotilapia ventralis	2	2	99.50	111.50	14.36	29.25	suction	aufwuchs	Yamaoka 1991
Simochromis babaulti	3	3	77.00	78.20	10.65	18.40	bite	algae	Yamaoka et al 1986, Yamaoka 1987
Spathodus erythrodon	3	1	59.50	38.25	7.30	15.50	bite	algae	Yamaoka et al 1986, Yamaoka 1987
Spathodus marlieri	2	4	67.17	44.67	8.17	16.50	bite	algae	Yamaoka et al 1986, Yamaoka 1987
Tanganicodus irsacae	2	2	53.75	40.50	6.59	19.25	bite	invertebrates, algae	Koning 1998
Triglachromis otostigma	2	4	77.50	45.50	9.81	16.25	suction	benthic prey	Koning 1998
Tropheus brichardi	1	3	93.00	133.50	14.24	19.00	bite	algae	Yamaoka et al 1986, Yamaoka 1987
Tropheus moorii	2	2	78.50	89.00	11.87	19.00	bite	algae	Yamaoka et al 1986, Yamaoka 1987
Xenotilapia flavipinnis	3	1	60.00	30.75	7.53	15.25	suction	benthic prey	Koning 1998
Xenotilapia ochrogenys	0	5	92.80	65.00	11.90	24.60	suction	benthic prey	Koning 1998
Xenotilapia spiloptera	0	6	82.33	58.67	10.40	21.80	suction	benthic prey	Koning 1998
